# Positive parenting for healthy living (Triple P) for parents of children with type 1 diabetes: protocol of a randomised controlled trial

**DOI:** 10.1186/s12887-016-0697-4

**Published:** 2016-09-22

**Authors:** Aditi Lohan, Amy E. Mitchell, Ania Filus, Kate Sofronoff, Alina Morawska

**Affiliations:** 1 Parenting and Family Support Centre, School of Psychology, The University of Queensland, Brisbane, QLD 4072 Australia; 2 Center for Self-Report Science, Center for Social & Economic Research, University of Southern California, Los Angeles, USA

**Keywords:** Child behaviour, Parenting, Parenting intervention, Protocol, Randomised controlled trial, Self-efficacy, Type 1 diabetes

## Abstract

**Background:**

Type 1 diabetes is a serious, life-long condition which causes major health, social and economic burden for children, their families and the community. Diabetes management involves strict adherence to a complex regimen, and poor management and non-adherence are a persistent problem among children. Parent-child interactions and parenting have been identified as crucial points of intervention to support children’s health and emotional well-being, yet few parenting interventions have been developed or evaluated for parents of young children. This paper describes a randomised controlled trial of a brief, group-based parenting intervention for parents of young children (2-10 years) with type 1 diabetes compared against care as usual (CAU).

**Methods/design:**

Families will be randomised to either Positive Parenting for Healthy Living Triple P or CAU. Positive Parenting for Healthy Living Triple P involves 2 × 2 h group sessions. Outcomes will be assessed via parent and child questionnaire, home observations and blood glucose monitoring at baseline, 1-month and 6-months post-intervention. Primary outcomes will be parent- and child-reported parenting behaviour, parent-reported child behaviour and adjustment, and parent-reported child quality-of-life. Secondary outcomes will include parental self-efficacy with diabetes management, illness-specific and general parenting stress, parent-reported child illness behaviour, family quality-of-life, observed parenting and child behaviour, and child’s illness control.

**Discussion:**

The theoretical background, study hypotheses, methods and planned analyses are discussed.

**Trial registration:**

Australian New Zealand Clinical Trials Registry: ACTRN12613001281785. Registered 20 November, 2013.

## Background

It is estimated that, on an annual basis, 70,000 children under 15 years of age develop type 1 diabetes worldwide [1], and the incidence is rising globally with an estimated overall annual increase of approximately 3 % [2]. Type 1 diabetes is a chronic condition which requires constant attention and monitoring. Day-to-day management involves frequent blood glucose monitoring, multiple insulin injections, and regulation of carbohydrate intake and physical activity to prevent short-term and long-term complications [3]. Despite the problems associated with poor management, non-adherence to diabetes treatment regimens is a persistent problem, and adherence tends to be worse than for other chronic health conditions [4]. Rates of adherence for insulin injections have been reported to range from 20 to 80 %, about 65 % for dietary recommendations, 57 to 70 % for blood glucose monitoring, and 19 to 30 % for adherence to exercise regimens [5]. Research has established links between poor illness management and children’s psychological health [6], and behaviour [7], which may in turn contribute to poorer diabetes control [8].

Parents play an important role in management of diabetes, especially with young children when parents assume complete responsibility for illness management. Parents of children with type 1 diabetes need to integrate general parenting tasks, such as helping children manage their emotions and providing them with appropriate social and play experiences, with diabetes-specific tasks, such as maintaining optimal blood glucose levels and responding quickly and effectively to emergency situations (e.g., extreme hypoglycaemia). Management of type 1 diabetes in young children requires them to cooperate with their treatment regimen, yet research has demonstrated that children with diabetes tend to report more behavioural, adjustment and emotional problems compared to healthy children [9–11].

The link between ineffective parenting and child behaviour problems [12–15] has been clearly established, and it plays an important role in parenting children with diabetes [16]. Positive parenting behaviours characterised by positive, confident and effective parenting have been associated with better management of diabetes [17], better child adjustment [17], and diabetes-related quality of life [18]. Parenting and family factors also play an important role in child health outcomes. Factors such as positive and confident parenting have been linked with good diabetes control and treatment adherence in children [17, 19]. On the other hand, inconsistent and ineffective parenting practices (for instance, permissive parenting and overprotection) have been associated with poor metabolic control and adherence [20, 21].

In spite of the clear links between parenting and child outcomes in the context of type 1 diabetes, a recent review highlights the scarcity of parenting interventions for this population [22]. Most existing interventions are educational in nature, focusing narrowly on the child’s medical management rather than on the broader psychosocial context [23–26], despite evidence that behavioural interventions are more effective than educational interventions [27]. Although data on effectiveness of parenting interventions in children with type 1 diabetes is limited due to a relative dearth of well-controlled studies in this area, existing parenting interventions have demonstrated some potential for effectiveness in improving responsibility sharing and child cooperation in diabetes management, child behaviour difficulties, parental behaviour, parents’ psychological distress and child health outcomes [22]. Interventions aimed at reducing family conflict and improving family communication have also led to improved adherence rates and health outcomes for adolescents with diabetes [28–30].

Given the increasing prevalence of the condition, the reported difficulties with illness management, the higher levels of child behavioural and emotional problems and the lack of evidence based interventions, there is a critical need to establish the efficacy of parenting interventions for parents of children with type 1 diabetes so that evidence-based parenting programs can be made available to families. Parenting interventions which aim to improve positive parenting practices may lead not only to better general child behavioural and emotional outcomes, but also better child health outcomes. Interventions which focus on parental skills, parenting efficacy and effective self-regulatory skills can assist parents to manage their child’s condition more effectively through developing better daily routines and encouraging their child’s involvement in illness management [31].

## Application of triple P in type 1 diabetes

The Triple P - Positive Parenting Program is an established, evidence-based system of parenting intervention [32]. It is based on social learning principles, cognitive-behavioural and developmental theory, and aims to treat and prevent child behavioural and emotional difficulties by improving the skills, knowledge and confidence of parents, improving communication between parents, and reducing parental stress [33, 34]. Several meta-analyses have provided solid evidence that the program is effective in improving child behaviour and parenting outcomes [35–38]. Triple P has been adapted for and found to be effective in different populations such as parents of children with a disability [39], Indigenous families [40], and parents going through a divorce [41], among others.

To date there have been limited evaluations of Triple P with parents of children with a chronic health condition. Westruppet al. (2014) evaluated a 10-session individually delivered program (Standard Triple P) for parents of children with type 1 diabetes, aged 4–12 years. Parents randomly allocated to the intervention condition reported improved parent mental health, parenting skills and family functioning at 3 months post-intervention, but no effects were seen on child mental health, child behaviour and glycaemic control at either 3- or 12-month follow-up. Subgroup analyses revealed that the program was effective in improving child behaviour in children with pre-existing internalizing and externalizing behaviour problems, with moderate-to-large effect sizes at 3 months post-intervention [42].

Another study by Doherty, Calam and Sanders [43] evaluated whether the Self-Directed Teen Triple P workbook (10 modules) plus a chronic illness tip sheet could reduce diabetes-related family conflict and parental stress in parents of adolescents (aged 11-17 years) with type 1 diabetes. Participants in the intervention group reported significantly improved diabetes-related family conflict problems after participating in Triple P compared to those in usual care. However, no significant difference was found between groups for parental stress [43].

In both of these studies, the content of the program was neither adapted nor tailored specifically for parents of children with type 1 diabetes or any other health condition (except for the addition of a chronic illness tip sheet in the study by Doherty et al. [43]), and this may be one reason why effects were seen on some outcomes but not others. Also, both used lengthy interventions (each lasting 10 sessions), which may be difficult to implement with this population considering that the day-to-day management of diabetes is in itself challenging and time-consuming. With this in mind, we set out to investigate whether a brief, tailored adaptation of Triple P designed for parents of children with a chronic health condition (*Positive Parenting for Healthy Living*) would be effective in improving outcomes for parents and children with type 1 diabetes.

*Positive Parenting for Healthy Living* [44] is a two-session group program, with each session lasting about two hours. A brief, tailored program may be beneficial for families by minimizing the time required to participate in the program, resulting in increased parent attendance [45]. Parents of children with a chronic health condition often report feelings of isolation and lack of social support [46–48]; thus, a group program may provide an opportunity to connect with, discuss and learn from experiences of other parents experiencing similar issues, while improving cost-efficiency. This program has already been evaluated in a randomised controlled trial with parents of children with asthma and/or eczema (Morawska A, Mitchell A, Burgess S, Fraser J: Randomised controlled trial of Triple P for parents of children with asthma or eczema: Effects on parenting and child behaviour, submitted), which compared intervention to care as usual at baseline, 3- and 6-month follow-up, and demonstrated significant improvements in use of effective parenting strategies, parent stress, general as well as illness-specific child behaviour problems, parents’ confidence with illness management, and health-related quality of life for parents and families, with moderate to large effect sizes. Clinically and statistically significant improvements in parent-reported asthma and eczema severity were also reported, and results support the need for further research examining the effect of parenting interventions on illness management and health outcomes for other chronic health conditions.

A program with a generic condition approach was chosen for this study because primary care and community health services are more likely to care for children with a range of chronic health conditions; thus, a program addressing the common issues and concerns across different health conditions (with examples tailored for specific condition groups) may be more helpful from a large-scale population dissemination perspective. This is the first randomized controlled trial to test the efficacy of the *Positive Parenting for Healthy Living* program for parents of children with type 1 diabetes. Given the scarcity of parenting interventions for this population, and demonstrated efficacy of the program in the asthma and eczema context, the program may have the potential to improve parent and child outcomes in the type 1 diabetes population.

## Aims and hypotheses

This study aims to use multi-informant assessment to test the efficacy of *Positive Parenting for Healthy Living* for parents of children with type 1 diabetes. Specifically, the primary outcomes will be: (i) parent self-reported parenting behaviour; (ii) child-reported parenting behaviour; (iii) parent-reported child behaviour and adjustment; and (iv) parent-reported child health-related quality-of-life. Secondary outcomes will be: (i) child’s metabolic control (as indicated by HbA1c and within-range blood glucose readings); (ii) parents’ self-efficacy with diabetes management; (iii) parent-reported illness-specific child behaviour problems; (iv) parent-reported family quality-of-life; (v) parents’ diabetes-related stress and (vi) general parenting stress; (vii) observed parenting behaviour; and (viii) observed child behaviour. We hypothesise that, compared to a CAU group, the intervention group (INT) will show significantly greater improvement on each outcome measure at (a) post-intervention and (b) 6-month follow-up.

## Method

### Approach and methodology

This research will evaluate a brief skills-training program for parents of children with type 1 diabetes in a randomised controlled trial comparing the parenting intervention against care as usual. A care as usual group was chosen as the comparator to allow for evaluation of the intervention against current practice. The CONSORT guidelines for randomised controlled trials will be used.

### Design

The study is a 2 (Triple P vs CAU) × 3 (time: pre-test, post-test, 6-month follow-up) design superiority trial with 1:1 allocation ratio.

### Ethics

Ethical approval has been obtained from the Queensland Children’s Health Services Human Research Ethics Committee (HREC/14/QRCH/1) and the University of Queensland Behavioural and Social Sciences Ethical Review Committee (2013001357). This trial has been registered with the Australian and New Zealand Clinical Trials registration: ACTRN12613001281785 (Appendix A: Table 2). Ethics amendments approval will be sought before any further modification to the protocol is made. Any further approved changes to the protocol will also be updated on the trial registry.

### Participants

Participants will be 60 families of 2–10 year old children with type 1 diabetes, recruited through primary care settings, paediatric specialists, specialist clinics at major Brisbane hospitals, and through targeted mail-out and media campaigns. Diagnostic confirmation of the child’s diabetes will be sought from the child’s treating diabetes team.

#### Inclusion and exclusion criteria

Parents of children aged 2-10 years with type 1 diabetes will be recruited. Parents must be concerned about their child’s behaviour, emotions, or illness management to be included in the study. Parents will be excluded if: (i) the child has a disability, including language and speech impairment; (ii) parents are currently seeing a professional for the child’s behaviour difficulties; (iii) the child has been diagnosed with type 1 diabetes for less than three months; (iv) parents are currently receiving psychological help or counselling; (v) parents are intellectually disabled; or (vi) parents do not read and understand English.

#### Recruitment

Participants will primarily be recruited through in-clinic recruitment at the Endocrinology Clinic of the Lady Cilento Children’s Hospital (Brisbane, Australia). All parents with a child between 2 and 10 years of age with a diagnosis of type 1 diabetes listed on the hospital database will be mailed a letter of invitation to participate in the study. Parents can register their interest in the study by phone, email, or on the study website. Parents will also be encouraged to discuss any questions or concerns regarding participation with a member of the research team who will be present in the clinic.

Recruitment brochures and posters will also be distributed and displayed at GP clinics and paediatrician offices across the Greater Brisbane area. In addition, information about the study will be emailed to Brisbane schools for inclusion in school newsletters. The same information will also be available through advertisements posted on websites and social media pages of relevant organisations, such as Diabetes Queensland and the Juvenile Diabetes Research Foundation, Australia. Other sources of recruitment include advertisements in the University of Queensland staff newsletter and posts on diabetes-specific parenting forums.

Interested parents will be assessed for eligibility and enrolled in the study by the study coordinator. Following eligibility screening, both parents will complete self-report assessment measures as relevant, and a home observation and child report measures will also be completed. All parent and child participants will receive information and consent forms detailing the project, and consent will only be gained once participants have had an opportunity to address any concerns or questions. Consent will also be sought to contact the family’s diabetes team to obtain confirmation of the child’s diagnosis and details of illness status. Participation will be completely voluntary and participants will be free to withdraw at any time. No further data will be collected from participants who actively withdraw from the study. Any families needing additional help at follow-up will be provided with appropriate referrals to community-based professional services. Participants will be provided with contact details for the research team and ethics committee should they need to report any adverse events. Detailed progress reports will be submitted to the relevant ethics committees annually, and any adverse events will be reported within 72 h.

#### Randomisation

Randomisation of participants to either the INT or CAU group will be done using a random allocation sequence, generated by a researcher not involved in the project, using a computer-based random number generator. A pre-prepared series of sealed opaque envelopes, each labelled with a participant ID number and containing a Randomisation Notification Letter, will be used to conceal the group allocation from researchers and participants until after completion of the baseline assessment. Participants in either group will continue to access their regular medical treatment with their child’s diabetes team.

Immediately after completion of the baseline home observation session, the research assistant conducting the home visit will open the envelope, and participants will be notified of their condition. The Randomisation Notification Letter will be provided to the participant to retain for their records.

**Fig. 1:**
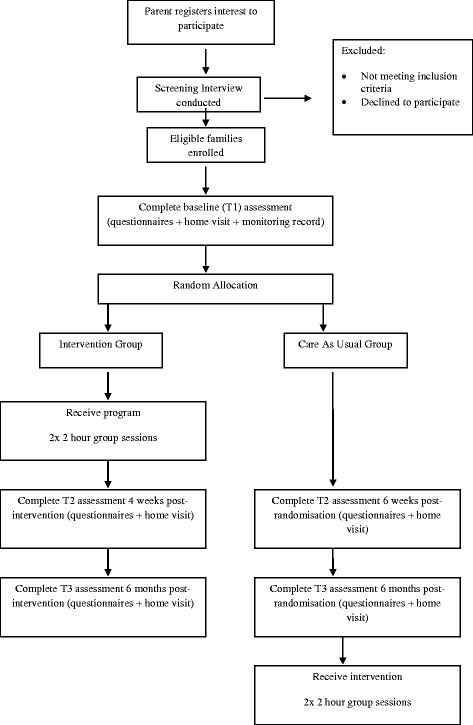
Participant flow through the study

Participants will be assigned to intervention sessions based on individual preferences for day, time, and location, depending on availability of groups. Participants will be assigned to intervention sessions as soon as possible after randomisation. The flow of participants through the study is summarised in Fig. 1.

Due to the nature of the intervention, neither participants nor research staff can be blinded to condition allocation, with the exception of coders who will code randomly-allocated video files of home observation sessions and remain blinded to participant allocation and assessment time-point. Since other research staff will not be blinded to allocation, a data monitoring committee is not needed.

### Intervention

The intervention will consist of two, 2-h *Positive Parenting for Healthy Living* group discussion sessions. The content of the sessions draws on the theoretical principles that form the basis of Triple P [32]. The sessions are designed to be interactive and provide opportunities for discussion.

The proximal targets of the intervention are parenting skills and confidence relating to both general child behaviour, and behaviour problems specific to illness management. The intervention aims to increase parental self-regulation, promote child self-regulation, increase positive parenting practices to promote child cooperation (particularly relating to illness management), lead to consistent discipline and promote routine, and enhance parents’ capacity to take care of themselves in order to reduce parenting stress and improve family wellbeing. The intervention will be conducted by parents with their children and there will be no direct contact with children in terms of intervention delivery.

#### Positive parenting for healthy living – part I

The first session is designed as an introduction to the principles of positive parenting in the context of child chronic illness management. It begins by exploring the impact of the child’s condition on the child, parent, and family, and introduces the principles of positive parenting as a way to promote children’s development and manage children’s behaviour and emotions in a constructive way.

It aims to assist parents to develop strategies to manage their child’s condition effectively while minimising the impact on the child and family by continuing regular activities, having realistic expectations of their child and themselves, involving the child in age-appropriate diabetes management tasks, reducing child and family stress, balancing work and family, working as a team, communicating effectively with the child’s diabetes team and caregivers, and helping siblings cope.

#### Positive parenting for healthy living – part II

The second session will be conducted one week after the first session. It aims to build on the principles of positive parenting introduced in Part I, to promote positive practices, assist parents to develop effective disciplinary methods, and help create environments conducive to caring relationships between parents and their children. It begins with a discussion of why children with chronic health conditions may be at risk of behaviour problems, before examining common parenting traps from the perspective of parenting a child with a chronic illness. The session focuses on providing parents with strategies that will empower them to prevent and manage problem behaviours and ensure their children are implementing their illness prevention and management plan appropriately (e.g., taking medication as prescribed, using devices correctly). Assertive discipline strategies are also discussed, including giving clear instructions, using praise effectively, and managing disobedience and problem behaviour.

Parents will also be provided with a ‘Communicating with Others’ tip sheet, developed for this study, outlining suggestions for how to effectively communicate and work together with the child’s other caregivers and health care professionals*.* Finally, parents will be encouraged to take their child to regular, ongoing visits with their diabetes team.

##### Care as usual condition

Families in CAU will complete assessments at baseline, 6 weeks later, and then again at 6 months. During this time, families will continue to receive regular medical management from their usual diabetes team, as appropriate. After the 6-month follow-up assessment, families will be offered participation in the intervention.

##### Protocol adherence

Each practitioner delivering the intervention will be trained using a standardised system of training and accreditation, designed to promote program use and fidelity. Practitioners deliver Triple P according to a standardised manual and follow treatment delivery protocols, and complete protocol adherence checklists for each session conducted. These will be reviewed and coded by a research assistant familiar with the protocols for adherence. Practitioners will receive regular clinical supervision. Group sessions will be videotaped and independently coded for protocol adherence, using structured session checklists. The inter-rater reliability (kappa) of this coding will be assessed for 25 % of videotaped sessions by a second rater.

### Assessment

Table 1 provides a summary of the assessment measures. Socioeconomic status (including income, occupation status, parent education), ethnic background, single parenting, and parent age, as well as child age, gender and health will be assessed using the *Family Background Questionnaire* (FBQ) [49]. One parent will complete this measure, but all other parent-report measures will be completed by both parents, where relevant.

**Table 1:** Summary of assessment measures

Domain of assessment	Measures
Socio-demographic	Family Background Questionnaire/FBQ [49]
Parenting efficacy	Self-Efficacy for Diabetes Scale/SED [57]
Child illness behaviour	Diabetes Behaviour Checklist/DBC [58]
Parenting behaviour (completed by both parent and child)	Alabama Parenting Questionnaire/APQ (Frick PJ: The Alabama Parenting Questionnaire, Unpublished)
Child behaviour & adjustment	Child Adjustment and Parent Efficacy Scale/CAPES [52]
Child quality of life	PedsQL4.0 Generic Core Scale [54]
Family quality of life	PedsQL Family Impact Module [59]
Parent adjustment & stress	Parent Experience of Child Illness Scale/PECIS [61]
	Parenting Stress Index-Short Form (PSI/SF) [60]
Illness severity (monitoring)	Blood glucose readings downloaded directly from the child’s blood glucose meter; HbA1c levels
Child & parent behaviour	Home Observation (Sanders MR, Le Grice B, Turner KMT: Mealtime observation schedule: An observer’s manual, Unpublished)
Program satisfaction	Parent Satisfaction Questionnaire [62]
	Child Satisfaction Questionnaire [64]

All parent-report measures will be provided in a written (online or printed) self-administered questionnaire format, and will take approximately thirty minutes to complete.

### Primary outcome measures

#### Parenting behaviour

##### Parent-report

The *Alabama Parenting Questionnaire –Parent report* (APQ-PR; Frick PJ: The Alabama Parenting Questionnaire, Unpublished) is a 42 item measure of parent-reported parenting style, assessing aspects of positive parenting (α = .80), involvement (α = .80), inconsistent discipline (α = .67), poor supervision (α = .67), and corporal punishment (α = .46). The typical frequency of each parenting behaviour is rated using a 5-point scale ranging from 1 (*Never*) to 5 (*Always*). The measure has demonstrated convergent validity with independent observations of parenting behaviour [50]. For the purpose of the present study, we will be excluding the ‘poor supervision’ subscale (10 items) because type 1 diabetes in young children requires ongoing supervision from parents, making these items less relevant for our participants. Thus, parents will complete a 32 item measure of parenting behaviour.

##### Child-report

Children aged 4 years and older will complete the printed *Alabama Parenting Questionnaire-Child Report* (APQ-CR; Frick PJ: The Alabama Parenting Questionnaire, Unpublished) at each assessment time point. The child-report contains 51 items (there are two parts to each of the involvement questions - one for mother involvement, and one for father involvement) measuring child-reported parenting style, assessing involvement of mothers (α =. 72) and fathers (α =. 83), aspects of positive parenting (α =. 74), inconsistent discipline (α = .56), poor supervision (α = .69), and corporal punishment (α = .44). The typical frequency of each parenting behaviour is rated using a 5-point scale ranging from 1 (*Never*) to 5 (*Always*). For reasons similar to the APQ-Parent Report, the ‘poor supervision’ subscale (10 items) will be excluded for the APQ-Child Report as well, resulting in a 41-item measure of parenting behaviour that children will complete. Children will complete this questionnaire during home visit sessions. The parent will not be in the room with the child during this segment. A member of the research team will remain in the room to provide assistance to the child in completing the questionnaire, as needed. The findings from this questionnaire will be interpreted with caution as the initial validation study revealed that young children (below 9 years) are more likely to respond using a consistent response set, either answering high or low on all items [50]. However, the child-report form has been used with young children to evaluate associations between parenting and child outcomes [51]. As children at different ages may interpret and understand questions in different ways, and since their perceptions may be influenced by the events of a particular day, their mood, and various other things happening around them, extra care will need to be taken when drawing inferences from child responses.

#### Child behaviour and adjustment

##### Parent-report

General child behaviour will be assessed using the *Child Adjustment and Parent Efficacy Scale* (CAPES) [52], a 30-item measure of parental perceptions of child behavioural and emotional adjustment. Parents rate each item from 0 (*Not true at all*) to 3 (*True most of the time*) depending on how true the statement was for their child in the past 4 weeks. Items are summed to yield a total intensity score (range of 0–90), a behaviour score (range of 0–78), and an emotional maladjustment score (range of 0–12), where higher scores indicate higher levels of problems. The Confidence Scale consists of 20 items and measures parents’ level of confidence in managing child emotional and behavioural problems. Parents rate each item from 1 (*Certain I can’t do it*) to 10 (*Certain I can do it*) depending on how confident they are in successfully dealing with their child’s behaviour. The possible range for this scale is 20–200, with higher scores indicating greater levels of parent efficacy. This measure has demonstrated good internal consistency for both Intensity (*α* = .90 and .74 for behavioural and emotional subscales, respectively) and Confidence (*α* = .96) scales [53].

#### Child’s quality of life

##### Parent-report

Children’s health-related quality of life will be assessed using the *PedsQL4.0: Pediatric Quality Generic Core Scale* [54], which is a 23-item measure assessing the core dimensions of health (physical, emotional, social and school functioning). Parents rate each item from 0 (*Never*) to 4 (*Almost always*) depending on how true the statement was for their child in the past month. Two summary scores (physical and psychosocial health) and a total score are calculated. Items are reverse-scored and transformed on a scale from 0-100 (0 = 100, 1 = 75, 2 = 50, 3 = 25, 4 = 0), and mean item scores are used to calculate summary and total scores. Higher scores indicate better quality of life. It has high internal consistency (α = .90), distinguishes between healthy children and children with acute and chronic health conditions, as well as disease severity within a chronic health condition, and is sensitive to change.

### Secondary outcome measures

#### Child’s illness severity and control

##### Parent-report

Parents will provide diabetes-specific information as well as information regarding the child’s illness severity and control at baseline, including the child’s age at diagnosis, the date and value of child’s most recent HbA1c, target blood glucose ranges (before meals, after meals, at bedtime and overnight), the current treatment regime (insulin/pump therapy/use of continuous blood glucose monitoring device), parent-child responsibility-sharing in diabetes management, and frequency and description of hospitalisations due to diabetes complications.

##### Monitoring

To obtain an indication of short-term metabolic control, parents will also be asked to provide a record of routine blood glucose readings (from the child’s blood glucose meter or a paper diary) for the last 28 days. The readings will be directly downloaded from the child’s blood glucose meter at each home visit session (via Diasend software: www.diasend.com/au/), and a copy of readings will be taken for those keeping a diary record. After the readings are downloaded, the researcher will ask the parent: *How do you think the child’s blood glucose readings in the last month compare with how his/her readings are normally? Was the last month fairly typical?* The researcher will make note of any circumstances which were not typical such as illness, school holidays, etc.

The blood glucose readings downloaded at the home visits will be checked against the target ranges for in- and out-of-range readings. This will provide us with an indication of whether the intervention was helpful in bringing the blood glucose values into the target range at 1-month post-intervention and 6-month follow-up.

##### Diabetes team report

The target blood glucose ranges for each child will also be obtained from their diabetes team at the time of their enrolment in the study, which will be used to check for in- and out-of-range readings. Glycosylated haemoglobin (HbA1c) levels will be obtained from the child’s diabetes team at the time of their routine medical review. HbA1c levels provide an indication of an individual’s average blood glucose concentration over the previous 3 months, and are considered the best marker of longer-term diabetes control. As a reference point, the HbA1c levels are usually between 4.0 to 6.0 % in individuals who do not have diabetes. In Australia, HbA1c targets for children and adolescents with type 1 diabetes are <7.5 % [55, 56]. HbA1c levels of 8.0 % or above may indicate that tighter control of blood glucose levels is required. Similar to blood glucose values, change in HbA1c values will provide us with an indication of whether the intervention has an effect on longer-term glycaemic control.

#### Diabetes self-efficacy

##### Parent-report

The *Self-Efficacy for Diabetes Scale* (SED) [57] will be used to assess parents’ self-efficacy for managing their child’s diabetes. It contains 22 parenting tasks associated with diabetes management, and parents rate their confidence with performing each task on a 5-point scale (1*-Very sure I can’t* to 5*-Very sure I can*), indicating how much they believe they can or cannot do what is asked *now*. The scale has demonstrated good internal consistency (α = .87).

#### Child illness behaviour

##### Parent-report

Child illness behaviour will be assessed using the *Diabetes Behaviour Checklist* (DBC) [58], which consists of 24 behaviours that parents of children with diabetes often have to manage. Parents rate each item on a 7-point scale, from 1 (*Not at all*) to 7 (*Very much*), depending on the extent to which behaviours have been a problem for them with their child in the past 4 weeks (Extent score). Parents also rate their self-efficacy (Confidence score) for successfully dealing with each behaviour on a 10-point scale, from 1 (*Certain I can’t do it*) to 10 (*Certain I can do it*). Both the Extent and Confidence scales have demonstrated excellent internal consistency, α = .93 and .98, respectively (Lohan A, Morawska A, Mitchell A: Parenting Challenges related to Diabetes Management: Validation of the Diabetes Behaviour Checklist, In preparation).

#### Family quality of life

##### Parent-report

Parent and family quality of life will be evaluated using the 36-item *PedsQL Family Impact Module* [59]. This measure encompasses six scales measuring parent self-reported functioning (physical, emotional, social and cognitive functioning, communication, worry), and two scales measuring parent-reported family functioning (daily activities and family relationships). This measure yields a total score (α = .97), and two summary scores [parent health-related quality of life (α = .96) and family functioning (α = .90)]. Parents rate each item from 0 (*Never*) to 4 (*Almost always*) depending on how true the statement was for them and their family in the past month. Similar to the *PedsQL Generic Core Scale,* items are reverse-scored and transformed on a scale from 0-100, with higher scores indicating better quality of life.

#### Parenting stress

##### Parent-report

General parenting stress will be evaluated using *The Parenting Stress Index/Short Form* (PSI/SF) [60], which is a 36-item self-report instrument designed to measure the relative magnitude of stress in a parent–child system and to identify the sources of stress. Parents respond to each statement using a 5-point scale (from *Strongly Agree* to *Strongly Disagree*), to indicate the degree to which that item describes their beliefs. The PSI yields three subscales, including Parental Distress, Parent Child Dysfunctional Interactions, and Difficult Child, as well as a Total Stress score. The PSI/SF is highly correlated with the full-length PSI instrument (*r* = .94), and the 2-week test–retest reliability of the full-length PSI with the PSI/SF is *r* = .95.

*The Parent Experience of Child Illness Scale* (PECIS) [61] is a 25-item self-report scale which assesses parents’ adjustment to the experience of their child’s chronic illness. Parents rate their thoughts and feelings over the past month on a 5-point scale from 0 (*Never)* to 4 (*Always*). This measure provides scores on four subscales: Emotional Resources (range 0–20), Long-Term Uncertainty (range 0–20), Guilt and Worry (range 0–44), and Unresolved Anger and Sorrow (range 0–32). This measure has acceptable reliability (ranging from α = .72–.89), and evidence of construct validity for all subscales.

#### Parenting and child behaviour

##### Home observation

After parents complete questionnaires, families will participate in a forty-minute home observation session at each time point, to provide an objective assessment of child and parent behaviours, and skill in illness management. The observation will consist of three segments: (i) a diabetes management segment, where the parent will provide or supervise the child’s regular illness management, i.e. check the child’s blood glucose level, and administer the child’s regular insulin injection or change the insulin pump site, as relevant; (ii) a mealtime observation segment, where the child will participate in a typical mealtime with their family; and (iii) completion of the *Alabama Parenting Questionnaire-Child Form* with the child.

Observations will be videotaped and coded by trained research assistants blind to group assignment. Inter-rater agreement will be calculated by having a random 25 % of videotapes coded by a second research assistant.

A modified version of the *Mealtime Observation Schedule* (MOS; Sanders MR, Le Grice B, Turner KMT: Mealtime observation schedule: An observer’s manual, Unpublished) will be used to code appropriate and inappropriate child and parent behaviours. Each behaviour is rated as present or absent in each 10-s interval. We are currently piloting this modified version of the MOS. In addition, for the diabetes management segment a global rating of parent-child interactional tone will be made on a 9-point scale from 1 (*Very positive*) to 9 (*Very negative*).

#### Program satisfaction measures

##### Parent-report

Following the intervention, parents will complete a 13-item *Client Satisfaction Questionnaire* [62], which measures the participants’ satisfaction with the services they received. Parents use a 7-point scale to rate 10 items assessing the quality of the service received, the extent to which the program met their own and their child’s needs, and how much the program helped the parents develop skills and improve their child’s behaviour. The item scores will be summed to generate a total score ranging from 10 to 70, with higher scores indicating greater program satisfaction. The rest of the items are posed as open-ended questions. This measure is an adaptation of the Therapy Attitude Inventory [63], which has demonstrated high internal consistency (*α* = .88) and discriminant validity.

##### Child-report

Following the intervention, children will complete a 6-item *Child Satisfaction Questionnaire* [64], which addresses questions about the extent to which the child was aware of their parents undertaking a parenting program, and any changes that the child noticed in their parents’ interactions with them since participating in the program. Children will rate five items: whether they feel they are getting along better with their parents, how well their parents manage misbehaviour, whether they think that their parents learnt useful things in the program, whether their parents have been saying more nice things to them after the program, and if they are pleased that their parents did the program, on a 5-point scale, with responses ranging from *No* to *Heaps more*. The item scores will be summed to generate a total score, with higher scores indicating greater child satisfaction.

### Data management

All research investigators and the research coordinator will have full access to the data collected, and will be jointly responsible for data collection, data entry, analysis and write-up of results. To maintain the confidentiality of data, all hard copy records, such as screening interview forms, questionnaires, and consent forms, will be kept in a locked filing cabinet in a locked office at the University. Data from printed and online questionnaires, as well as blood glucose data, will be entered into a computer file. Digital recordings of home visit data will be stored in a computer file and backed up to external hard drives. Data in computer files will be kept in password-protected files within the University computer network. Security with regard to online questionnaires will be maintained by the University. Data collected from participants will be stored in a re-identifiable format for 7 years after completion of the project in accordance with ethics committee requirements. The chief investigators of the study will have access to the final trial dataset, and any request to access de-identified data will go through the chief investigators. Any publications arising from this research will be devoid of any identifying information and results will be reported in an aggregate form only. Full study protocol will be published in a peer-reviewed journal.

### Statistical analyses

Prior to conducting the main analyses, data will be screened for distributional assumptions (e.g., univariate and multivariate normality, outliers, and multi-collinearity) as well as inter- and intra-measure consistency. Preliminary analyses will also investigate whether the study groups differ on any demographic or clinical characteristics at baseline. Group differences in baseline characteristics will be examined via linear (for continuous outcomes) and logistic (for categorical outcomes) regression models. It is anticipated that the groups will not differ at baseline due to randomisation to study conditions. If baseline group differences are detected, we will investigate and report the extent to which the results from the planned analyses described below are altered, when these differences are statically controlled. For missing data points, an analysis of missing data will be conducted (see paragraph below on missing data) and imputation methods will be considered [65]. Data analysis will follow the intention-to-treat principle, which means that the study population subjected to the analysis will consist of all randomised individuals.

### Primary analyses

The primary analyses will evaluate the effects of the intervention on parent and child outcomes. These analyses will include reports obtained from parents who were the primary receivers of the intervention as well as reports on child’s illness severity and observational assessment. A multilevel modeling approach will be used to take into account the repeated measurements and thus non-independence of observations [66]. Dummy codes contrasting the groups will serve as fixed effects, allowing for random intercepts and slopes to vary across individuals. Significant fixed effects contrasting INT with CAU will indicate whether the intervention is an improvement over usual care. Separate models will be estimated for each outcome measure using Bonferroni correction to control for inflation of Type 1 error due to multiple comparisons.

### Secondary analysis

The secondary analysis will take advantage of the multiple-informant design of the study. We will investigate if the effects of the intervention are the same for parents who attended the intervention as opposed to their partners or their children, with respect to parenting practices, child adjustment, parental self-efficacy (general and diabetes management related), family quality of life and parental stress. We will apply a multilevel modeling approach adopted for dyads (mother and father) or triads (mother, father, and child) [67, 68] to model change in outcome variables for mothers, fathers and children individually while accounting for similarities within dyads or triads. We will investigate whether the intervention is beneficial for both parents, or has different effects for a parent who participated in the intervention as opposed to one that didn’t. Further, we will examine if the changes in parenting practices are the same as reported by mother, father and the child. Finally, we will evaluate whether the intervention has an effect on the family as a whole, such as decreasing discrepancies between mothers and fathers.

### Sample size and power analysis

The required sample size for the study was calculated to assure 80 % power to detect an effect size of ES = .5 for a mean difference in rates of change in the variables of interest between the groups (INT vs CAU and/or mother vs father vs child). This effect size is categorized as medium based on Cohen’s guidelines and has been chosen based on our previous research [35]. In multilevel models with repeated measures, the sample size is effectively the number of observations (the level-1), not number of participants (level-2 units). We performed power analysis using G*Power software [69] for rANOVA, looking at the within-between interactions and allowing for conservative estimate of intra-individual variability (.5). The analyses indicated that a sample size of 50 is sufficient to detect an ES of .5 at the significance level of .05 (two-tailed). This is without taking into account the added power accorded by the rich repeated measures in our data. Assuming 16 % attrition rate, an available sample of 60 families will be sufficient to detect medium sized effects.

### Missing outcome data

In any longitudinal design it is inevitable that some individuals will drop out from the study. For this reason, the study sample size was calculated to allow for a dropout rate of 16 %. Assuming that the complete data may only be available for 84 % of families at the end of the study, intention-to-treat analysis will be applied to allow all randomized families to be included in the analyses. Missing data will be accommodated via implementation of full information maximum likelihood analysis (FIML). This approach yields intention-to-treat estimates consistent with what would be expected if there were no missing data, given that the assumptions of either Missing Completely at Random (MCAR) or Missing at Random (MAR) are met [65].

## Discussion

This protocol paper outlines the background and design of a randomised controlled trial of *Positive Parenting for Healthy Living* for parents of children with type 1 diabetes. This study will be the first to trial this version of Triple P program in this population, and will help address the dearth of well-controlled parenting intervention research for parents of children with type 1 diabetes. This project will employ a rigorous methodology, with multi-domain and multi-informant assessment in order to inform future intervention development and tailoring. In this study, we are taking a novel approach in shifting the focus of diabetes management from a traditional medical management approach to a more holistic approach which includes parenting intervention as a component of an illness management plan. In addition, including perceptions of intervention outcomes of both parents and children and applying an advanced statistical approach to modelling family system data will assist in expanding the theoretical understanding of outcomes and tailoring the intervention to better ensure sustainability of outcomes, as eventually it is the children that are the target and assumed beneficiaries of parenting programs. However, since this area is still in infancy and there is a paucity of well-validated child assessment measures for young children, it raises certain methodological concerns, such as the validity and interpretation of child responses. Thus, special care and caution will need to be exercised when interpreting child responses in this study.

Recruitment and enrolment to the study commenced in April 2014, and will continue until mid/late 2016. Results will be published in a peer-reviewed journal when the data collection and analyses are complete. It is expected that participating in a brief, group-based parenting intervention has the potential to decrease ineffective parenting behaviour, improve general child and illness behaviour, improve child and family quality of life, reduce parent stress, enhance parental self-efficacy and improve child’s illness control.

## Appendix A

**Table 2:** Items from the World Health Organization Trial Registration Data Set

Data category	Information
Primary registry and trial identifying number	Australian New Zealand Clinical Trials Registry: ACTRN12613001281785
Date of registration in primary registry	20 November 2013
Secondary identifying numbers	N/A
Source(s) of monetary or material support	Australian Research Council’s (ARC) *Discovery Projects* scheme
	(Project ID: DP140100781)
Primary sponsor	N/A
Secondary sponsor(s)	N/A
Contact for public queries	Dr Alina Morawska
Contact for scientific queries	Dr Alina Morawska
Public title	Efficacy of the Positive Parenting Program (Triple P) for parents of young children with type 1 diabetes
Scientific title	Efficacy of the Positive Parenting Program (Triple P) for parents of children with type 1 diabetes in improving parenting skills and confidence relating to general child behaviour and illness management?
Countries of recruitment	Australia
Health condition(s) or problem(s) studied	Type 1 diabetes (Illness severity/control)
	Parenting practices/behaviour
	Child behaviour and adjustment
	Parent adjustment and stress
	Child illness behaviour
	Parenting efficacy
	Child's quality of life
	Family quality of life
Intervention(s)	2 × 2 h Positive Parenting for Healthy Living group discussion sessions
Key inclusion and exclusion criteria	The key inclusion criteria is: (i) presence in the family of a 2-10 year old child; and (ii) child has diagnosis of type 1 diabetes.
	The exclusion criteria include: (i) child has a disability including language and speech impairment; (ii) parents are currently seeing a professional for the child’s behaviour difficulties; (iii) parents are currently receiving psychological help or counselling; (iv) parents have difficulties in reading a newspaper or (v) the child has been diagnosed in the last three months.
Study type	RCT, 2 × 3 design, superiority trial, 1:1 allocation ratio
Date of first enrolment	May 2014
Target sample size	60
Recruitment status	Recruiting
Primary outcome(s)	Parenting practices/behaviour
	Child behaviour and adjustment
	Child’s quality of life
Key secondary outcomes	Illness severity and control
	Parent adjustment and stress
	Child illness behaviour
	Parenting efficacy
	Family quality of life

## Abbreviations

APQ-CR: Alabama parenting questionnaire –Child report
APQ-PR: Alabama parenting questionnaire –Parent report
CAPES: Child adjustment and parent efficacy scale
CAU: Care as usual
CONSORT: Consolidated standards of reporting trials
DBC: Diabetes behaviour checklist
ES: Effect size
FBQ: Family background questionnaire
FIML: Full information maximum likelihood analysis
HbA1C: Glycosylated haemoglobin
ID: Identification
INT: Intervention
MAR: Missing at random
MCAR: Missing completely at random
MOS: Mealtime observation schedule
PECIS: Parent experience of child illness scale
PSI/SF: Parenting stress index-short form
SED: Self-efficacy for diabetes scale
Triple P: Positive parenting program

## References

[1] International Diabetes Federation. IDF Diabetes Atlas, 3rd edn. Brussels: International Diabetes Federation; 2006. Available at: http://www.diabetesatlas.org/resources/previous-editions.html. Accessed 1 July 2015.

[2] Patterson CC, Dahlquist GG, Gyürüs E, Green A, Soltész G (2009). Incidence trends for childhood type 1 diabetes in Europe during 1989–2003 and predicted new cases 2005–20: a multicentre prospective registration study. Lancet.

[3] Diabetes Control and Complications Trial Research Group (DCCT) (1994). Effect of intensive diabetes treatment on the development and progression of long-term complications in adolescents with insulin-dependent diabetes mellitus: Diabetes Control and Complications Trial. J Pediatr.

[4] DiMatteo MR (2004). Variations in patients’ adherence to medical recommendations: A quantitative review of 50 years of research. Med Care.

[5] McNabb WL (1997). Adherence in diabetes: Can we define it and can we measure it?. Diabetes Care.

[6] Hood KK, Huestis S, Maher A, Butler D, Volkening L, Laffel LMB (2006). Depressive symptoms in children and adolescents with type 1 diabetes: association with diabetes-specific characteristics. Diabetes Care.

[7] McDonnell CM, Northam EA, Donath SM, Werther GA, Cameron FJ (2007). Hyperglycemia and externalizing behavior in children with type 1 diabetes. Diabetes Care.

[8] Fisher E, Delamater AM, Bertelson AD, Kirkley BG (1982). Psychological factors in diabetes and its treatment. J Consult Clin Psychol.

[9] Cohen DM, Lumley MA, Naar-King S, Partridge T, Cakan N (2004). Child behavior problems and family functioning as predictors of adherence and glycemic control in economically disadvantaged children with type 1 diabetes: a prospective study. J Pediatr Psychol.

[10] Castro D, Tubiana-Rufi N, Moret L, Fombonne E, PEDIAB Collaborative Group (2000). Psychological adjustment in a French cohort of type 1 diabetic children. Diabetes and Metabolism.

[11] Hilliard ME, Monaghan M, Cogen FR, Streisand R (2010). Parent stress and child behaviour among young children with type 1 diabetes. Child Care Health Dev.

[12] Guajardo NR, Snyder G, Petersen R (2009). Relationships among parenting practices, parental stress, child behaviour, and children’s social-cognitive development. Infant Child Dev.

[13] Snyder J, Cramer A, Afrank J, Patterson GR (2005). The contributions of ineffective discipline and parental hostile attributions of child misbehavior to the development of conduct problems at home and school. Dev Psychol.

[14] Shaw DS, Owens EB, Giovannelli J, Winslow EB (2001). Infant and toddler pathways leading to externalizing disorders. J Am Acad Child Adolesc Psychiatry.

[15] Arnold DS, O'Leary SG, Wolff LS, Acker MM (1993). The Parenting Scale: A measure of dysfunctional parenting in discipline situations. Psychol Assess.

[16] Armstrong B, Mackey ER, Streisand R (2011). Parenting behavior, child functioning, and health behaviors in preadolescents with type 1 diabetes. J Pediatr Psychol.

[17] Davis CL, Delamater AM, Shaw KH (2001). Brief report: Parenting styles, regimen adherence, and glycemic control in 4- to 10-year-old children with diabetes. J Pediatr Psychol.

[18] Botello-Harbaum M, Nansel T, Haynie DL, Iannotti RJ, Simons-Morton B (2008). Responsive parenting is associated with improved type 1 diabetes-related quality of life. Child Care Health Dev.

[19] Anderson BJ, Miller JP, Auslander WF, Santiago JV (1981). Family characteristics of diabetic adolescents: relationship to metabolic control. Diabetes Care.

[20] Shorer M, David R, Schoenberg-Taz M, Levavi-Lavi I, Phillip M, Meyerovitch J (2011). Role of parenting style in achieving metabolic control in adolescents with type 1 diabetes. Diabetes Care.

[21] Miller-Johnson S, Emery RE, Marvin RS, Clarke W, Lovinger R, Martin M (1994). Parent-child relationships and the management of insulin-dependent diabetes mellitus. J Consult Clin Psychol.

[22] Lohan A, Morawska A, Mitchell A (2015). A systematic review of parenting interventions for parents of children with type 1 diabetes. Child Care Health Dev.

[23] Fogel NR, Weissberg-Benchell J (2010). Preventing poor psychological and health outcomes in pediatric type 1 diabetes. Curr Diab Rep.

[24] McBroom LA, Enriquez M (2009). Review of family-centered interventions to enhance the health outcomes of children with type 1 diabetes. Diabetes Educ.

[25] Northam E, Todd S, Cameron F (2006). Interventions to promote optimal health outcomes in children with Type 1 diabetes—are they effective?. Diabet Med.

[26] Padgett D, Mumford E, Hynes M, Carter R (1988). Meta-analysis of the effects of educational and psychosocial interventions on management of diabetes mellitus. J Clin Epidemiol.

[27] Cushing CC, Steele RG (2012). A meta-analytic review of eHealth interventions for pediatric health promoting and maintaining behaviors. J Pediatr Psychol.

[28] Laffel LMB, Vangsness L, Connell A, Goebel-Fabbri A, Butler D, Anderson BJ (2003). Impact of ambulatory, family-focused teamwork intervention on glycemic control in youth with type 1 diabetes. J Pediatr.

[29] Satin W, La Greca AM, Zigo MA, Skyler JS (1989). Diabetes in adolescence: effects of multifamily group intervention and parent simulation of diabetes. J Pediatr Psychol.

[30] Wysocki T, Harris MA, Greco P, Bubb J, Danda CE, Harvey LM, White NH (2000). Randomized, controlled trial of behavior therapy for families of adolescents with insulin-dependent diabetes mellitus. J Pediatr Psychol.

[31] Morawska A, Calam R, Fraser J (2014). Parenting interventions for childhood chronic illness: A review and recommendations for intervention design and delivery. J Child Health Care.

[32] Sanders MR (2012). Development, evaluation, and multinational dissemination of the Triple P-Positive Parenting Program. Annu Rev Clin Psychol.

[33] Sanders MR (1999). Triple P-Positive Parenting Program: Towards an empirically validated multilevel parenting and family support strategy for the prevention of behavior and emotional problems in children. Clin Child Fam Psychol Rev.

[34] Sanders MR, Turner KM, Markie-Dadds C (2002). The development and dissemination of the Triple P-Positive Parenting Program: A multilevel, evidence-based system of parenting and family support. Prev Sci.

[35] Sanders MR, Kirby JN, Tellegen CL, Day JJ (2014). Towards a public health approach to parenting: A systematic review and meta-analysis of the Triple P-Positive Parenting Program. Clin Psychol Rev.

[36] De Graaf I, Speetjens P, Smit F, de Wolff M, Tavecchio L (2008). Effectiveness of the Triple P Positive Parenting Program on behavioral problems in children: a meta-analysis. Behav Modif.

[37] De Graaf I, Speetjens P, Smit F, De Wolff M, Tavecchio L (2008). Effectiveness of the triple P positive parenting program on parenting: a meta- analysis. Fam Relat.

[38] Nowak C, Heinrichs N (2008). A comprehensive meta-analysis of triple P-positive parenting program using hierarchical linear modeling: effectiveness and moderating variables. Clin Child Fam Psychol Rev.

[39] Whittingham K, Sofronoff K, Sheffield J, Sanders MR (2008). Stepping Stones Triple P: An RCT of a parenting program with parents of a child diagnosed with an Autism Spectrum Disorder. J Abnorm Child Psychol.

[40] Turner KMT, Richards M, Sanders MR (2007). Randomised clinical trial of a group parent education programme for Australian Indigenous families. J Paediatr Child Health.

[41] Stallman HM, Sanders MR (2014). A randomized controlled trial of family transitions triple P: a group-administered parenting program to minimize the adverse effects of parental divorce on children. J Divorce & Remarriage.

[42] Westrupp E, Northam E, Lee K, Scratch S, Cameron F (2014). Reducing and preventing internalizing and externalizing behavior problems in children with type 1 diabetes: a randomized controlled trial of the Triple P-Positive Parenting Program. Pediatr Diabetes.

[43] Doherty FM, Calam R, Sanders MR (2013). Positive parenting program (triple P) for families of adolescents with type 1 diabetes: a randomized controlled trial of self-directed teen triple P. J Pediatr Psychol.

[44] Morawska A, Sanders MR (2011). Positive Parenting for Healthy Living Parent Discussion Group.

[45] Sassmann H, de Hair M, Danne T, Lange K. Reducing stress and supporting positive relations in families of young children with type 1 diabetes: a randomized controlled study for evaluating the effects of the DELFIN parenting program*.* BMC Pediatrics. 2012;12. doi:10.1186/1471-2431-12-152. 10.1186/1471-2431-12-152 PMC3512538 22994843

[46] Gannoni AF, Shute RH (2010). Parental and child perspectives on adaptation to childhood chronic illness: A qualitative study. Clin Child Psychol Psychiatry.

[47] Sullivan-Bolyai S, Deatrick J, Gruppuso P, Tamborlane W, Grey M (2002). Mothers’ experiences raising young children with type 1 diabetes. J Specialists Pediatr Nurs.

[48] Landolt MA, Ribi K, Laimbacher J, Vollrath M, Gnehm HE, Sennhauser FH (2002). Posttraumatic stress disorder in parents of children with newly diagnosed type 1 diabetes. J Pediatr Psychol.

[49] Sanders MR, Morawska A (2010). Family Background Questionnaire.

[50] Shelton KK, Paul JF, Wootton J (1996). Assessment of parenting practices in families of elementary school-age children. J Clin Child Psychol.

[51] Frick PJ, Christian RE, Wootton JM (1999). Age trends in the association between parenting practices and conduct problems. Behav Modif.

[52] Morawska A, Sanders MR (2010). Child Adjustment and Parent Efficacy Scale (CAPES).

[53] Morawska A, Sanders MR, Haslam D, Filus A, Fletcher R (2014). Child adjustment and parent efficacy scale (CAPES): development and initial validation of a parent report measure. Aust Psychol.

[54] Varni JW, Seid M, Kurtin PS (2001). The PedsQL™ 4.0: Reliability and validity of the Pediatric Quality of Life Inventory™ Version 4.0 Generic Core Scales in healthy and patient populations. Med Care.

[55] APEG (Australasian Paediatric Endocrine Group). Clinical Practice Guidelines: Type 1 diabetes in children and adolescent*s*. 2005. Available at: https://www.nhmrc.gov.au/_files_nhmrc/publications/attachments/cp102.pdf. Accessed 1 July 2015.

[56] Rewers M, Pihoker C, Donaghue K, Hanas R, Swift P, Klingensmith GJ (2009). Assessment and monitoring of glycemic control in children and adolescents with diabetes. Pediatr Diabetes.

[57] Streisand R, Swift E, Wickmark T, Chen R, Holmes CS (2005). Pediatric parenting stress among parents of children with type 1 diabetes: The role of self efficacy, responsibility, and fear. J Pediatr Psychol.

[58] Morawska A, Mitchell A, Pay R (2012). Diabetes Behaviour Checklist.

[59] Varni JW, Sherman SA, Burwinkle TM, Dickinson PE, Dixon P (2004). The PedsQL family impact module: preliminary reliability and validity. Health Qual Life Outcomes.

[60] Abidin RR (1995). Parenting Stress Index.

[61] Bonner MJ, Hardy KK, Guill AB, McLaughlin C, Schweitzer H, Carter K (2006). Development and validation of the parent experience of child illness. J Pediatr Psychol.

[62] Sanders MR, Markie-Dadds C, Turner KMT (2001). Practitioner's manual for Standard Triple P.

[63] Eyberg SM (1993). Consumer satisfaction measures for assessing parent training programs. Innovations in Clinical Practice: A Source Book.

[64] Sofronoff K, Morawska A (2014). Child Satisfaction Questionnaire (CSQ).

[65] Enders CK (2010). Applied missing data analysis.

[66] Maas CJM, Hox JJ (2005). Sufficient sample sizes for multilevel modeling. Methodology: European Journal of Research Methods for the Behavioral and Social Sciences.

[67] Lyons KS, Sayer AG (2005). Longitudinal dyad models in family research. J Marriage Fam.

[68] Raudenbush SW, Brennan RT, Barnett RC (1995). A multivariate hierarchical model for studying psychological change within married couples. J Fam Psychol.

[69] Faul F, Erdfelder E, Buchner A, Lang AG (2009). Statistical power analyses using G*Power 3.1: Tests for correlation and regression analyses. Behav Res Methods.

